# Rock Crack Recognition Technology Based on Deep Learning

**DOI:** 10.3390/s23125421

**Published:** 2023-06-08

**Authors:** Jinbei Li, Yu Tian, Juan Chen, Hao Wang

**Affiliations:** 1 School of Hydraulic Engineering, Dalian University of Technology, Dalian 116024, China; lijinbei@mail.dlut.edu.cn (J.L.);; 2 Department of Water Resources Research, China Institute of Water Resources and Hydropower Research, Beijing 100038, China

**Keywords:** object detection, YOLOv7, attention, disaster, crack

## Abstract

The changes in cracks on the surface of rock mass reflect the development of geological disasters, so cracks on the surface of rock mass are early signs of geological disasters such as landslides, collapses, and debris flows. To research geological disasters, it is crucial to swiftly and precisely gather crack information on the surface of rock masses. Drone videography surveys can effectively avoid the limitations of the terrain. This has become an essential method in disaster investigation. This manuscript proposes rock crack recognition technology based on deep learning. First, images of cracks on the surface of a rock mass obtained by a drone were cut into small pictures of 640 × 640. Next, a VOC dataset was produced for crack object detection by enhancing the data with data augmentation techniques, labeling the image using Labelimg. Then, we divided the data into test sets and training sets in a ratio of 2:8. Then, the YOLOv7 model was improved by combining different attention mechanisms. This study is the first to combine YOLOv7 and an attention mechanism for rock crack detection. Finally, the rock crack recognition technology was obtained through comparative analysis. The results show that the precision of the improved model using the SimAM attention mechanism can reach 100%, the recall rate can achieve 75%, the AP can reach 96.89%, and the processing time per 100 images is 10 s, which is the optimal model compared with the other five models. The improvement is relative to the original model, in which the precision was improved by 1.67%, the recall by 1.25%, and the AP by 1.45%, with no decrease in running speed. This proves that rock crack recognition technology based on deep learning can achieve rapid and precise results. It provides a new research direction for identifying early signs of geological hazards.

## 1. Introduction

Devastating natural hazards such as landslides are massive threats to lives and property around the globe, especially in mountainous regions [1]. In recent years, due to the intensification of climate change, extreme weather has occurred more frequently, providing favorable conditions for landslides [2]. The Guangdong–Hong Kong–Macao Greater Bay Area [3] is prone to storm surges, heavy rain, floods, and their derivative geological disasters. With climate change and high urbanization, natural disasters in the Guangdong–Hong Kong–Macao Greater Bay Area are becoming more interchangeable and transitive, with complex mechanisms and great harm. From 2014 to 2020, there were 1446 landslide and collapse disaster points in the Guangdong–Hong Kong–Macao Greater Bay Area, most of which were distributed in the transition zone between hilly and shallow mountainous areas and plains, and where human engineering activities were very strong. Under the comprehensive influence of its complex natural geographical environment, climatic conditions, and human activities, mountain disasters such as collapses, landslides, and mudslides in the Greater Bay Area are frequent.

Complete rock masses undergoing weathering and geological reactions can lead to a decrease in strength and the formation of tiny cracks. Root cracks and ice cracks occur, which increase the development of rock cracks. After the crack is formed, the strength of the rock is further reduced. On the one hand, this leads to the crack and decomposition of the incomplete rock, creating source conditions for collapse, landslides, and debris flows, and on the other hand, it creates weak surfaces to causing dynamic conditions for collapse, landslides, and debris flows. Fatigue cracking is one of the most common failures in various types of load-bearing structures [4]. The development of cracks can lead to geological hazards. Therefore, cracks in a rock mass can be early signs of a geological disaster. Therefore, obtaining disaster information is of greater significance for disaster prevention and control.

The are many disaster identification methods. Yang Xiao et al. proposed a real-time identification method for urban storm disasters using Weibo data [5]. Chen Cao et al. used SBAS–InSAR for landslide identification [6]. Yuhui Jin et al. proposes SA-MFNet to achieve pixel-wise landslide detection based on multisource data fusion analysis [7]. The formation environment of geological disasters in this region is relatively complex. These methods are poorly time sensitive and subject to many limitations. At many survey sites, geological hazard experts cannot physically reach the scene and need to take a large number of photos of the scene with drones and use these pictures to study and judge the disaster. The number of images taken by drones is huge, and manual processing is time-consuming and laborious. Therefore, we considered using deep learning technology to pre-process the scene pictures taken by drones to save time and improve efficiency.

Thanks to the efforts of many researchers in computer technology, artificial intelligence has made significant progress. The application of computer vision in production and life has been very extensive, especially in object detection technology. In general, object detection methods include two-stage and one-stage detection [8]. Two-stage detection prioritizes quality, e.g., the Region with CNN feature (RCNN) series of methods, including RCNN [9], Fast-RCNN [10], Faster-RCNN [11], Mask-RCNN [12], Cascade-RCNN [13], and Grid-RCNN [14]. One-stage detection methods prioritize speed, e.g., You Only Look Once (YOLO) [15], YOLOv2 [16], YOLOv3 [17], YOLOv4 [18], YOLOv5, and Single-Shot MultiBox Detector (SSD) [19]. These methods have poor detection results when there are multiple objects densely arranged next to each other. YOLOv7 [20] is optimized for model structure re-participation and dynamic tag allocation. It is not only quick but also provides quality.

Presently, computer vision has many applications in the identification of cracks on concrete surfaces. LI Xiang et al. aimed to address the current situation of low efficiency and low precision in crack identification in concrete structures in civil engineering; a new crack identification method based on single-shot multi-box detection (SSD) was proposed based on deep learning theory [21]. To achieve the fast and precise location of bridge cracks, the effects of interference factors such as light variations and stain shadows were taken into account, and a bridge crack detection method combining a bridge inspection machine and improved YOLOv3 was proposed by Yang Fuqiang et al. [22]. YOLOv7 has been applied in multiple scenarios. Jianfeng Zheng et al. proposed an improved YOLOv7 model to improve insulator–defect detection [23]. Sun Y. X. et al. proposed a method for classifying and locating surface defects in hot-rolled strip steel based on the YOLOv7 target detection model [24]. Currently, object detection is rarely used to detect cracks in rock masses. To track and predict the development of rock surface cracks to avoid the occurrence of some geotechnical disasters and improve the detection rate of rock surface fissures, a method for tracking and predicting rock fissure development based on an improved Faster-RCNN algorithm was devised by Huang Xiaohong et al. [25]. The feature maps extracted by Faster-RCNN are single layer, the resolution is relatively low, and RoI Pooling brings a loss of precision due to rounding twice. However, there is no research on rock mass crack detection in images using the YOLOv7 algorithm. To further increase the research on the object detection method in the detection of rock mass cracks, this paper introduces the latest object detection method, YOLOv7, in the detection of rock mass cracks after improvement. As a new member of the YOLO family, the YOLOv7 balances speed and precision better than all known object detectors. The attention mechanism enables the model to focus on key regions to improve model performance. In this study, multiple attention mechanisms were inserted between the backbone networks of YOLOv7. The YOLOv7 method was combined with different attention mechanisms to improve the efficiency of crack detection. The model inserted by the attention mechanism refines the multidimensional features of the image so that the precision, recall, and AP of the model are improved to various degrees.

## 2. Materials and Methods

### 2.1. Study Area

The Guangdong–Hong Kong–Macao Greater Bay Area city cluster is located in the south of the Nanling Mountain Range and south of the South China Sea, and has a latitude and longitude position of 111°59′ E~115°28′ E, 21°56′ N~24°51′ N. The topography and landforms of the region are mainly terraces, hills, and plains. The interaction between ocean, land, and atmosphere is strong, and the climate is complex and changeable, with an average annual temperature of 22 °C and an average yearly rainfall of 2300 mm. The combination of climatic and topographic conditions in this region has made the Guangdong–Hong Kong–Macao Greater Bay Area a geological-disaster-prone area.

### 2.2. Data Collection and Preprocessing

The image data used in this study were obtained via UAV photography. The richness and complexity of the dataset helps with the iterative optimization of convolutional neural models. Rock cracks with different postures can help the model increase learning efficiency and avoid overfitting the model. Therefore, we used drones to shoot the same rock crack from multiple postures and angles, increasing the number of datasets on the one hand and increasing the number of features of the dataset on the other. This increases the performance of the model in identifying objects. The images captured by the UAV were cropped to 640 × 640.

**Figure 1:**
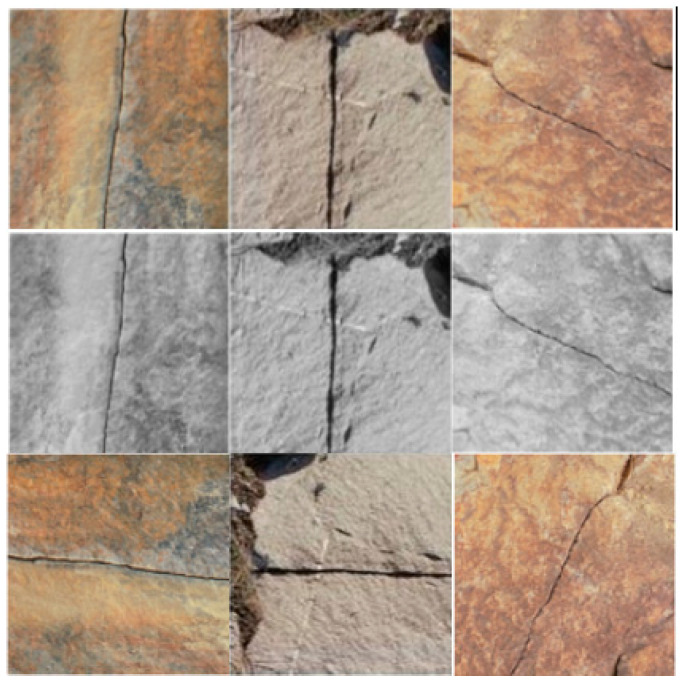
Crack image enrichment dataset.

In this study, many pictures were obtained using UAVs to photograph in the field. In this study, 200 images with different morphologies and complex backgrounds were selected from a large number of images to improve the representativeness of the sample data. To avoid model overfitting, the dataset was grayscale processed. The dataset was enriched with methods such as angle change. The expanded dataset was increased from 200 to 714 images. The expanded dataset is shown in Figure 1.

Then, this study used Labelimg to annotate the images and obtain the VOC dataset for cracked object detection. Finally, the data were divided into test and training sets in a ratio of 2:8 for training and testing.

### 2.3. Detection Principle

#### 2.3.1. YOLOv7

YOLOv7 is a new version of the object detection algorithms proposed by the YOLOv4 team. In order to accomplish a balance and enhancement in speed and precision, YOLOv7 has been meticulously designed in terms of model network structure and extensive parameterization.

**Figure 2:**
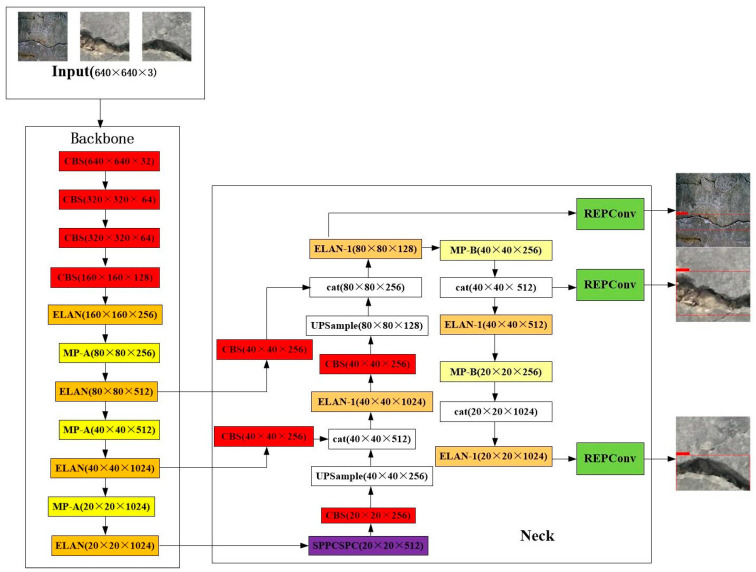
The structure of YOLOv7.

The network structure of the YOLOv7 object detection model comprises an input module, a backbone module, and a neck module.The flow chart of the model is shown in Figure 2 The input module employs the same data enhancement methods as YOLOv5, including Mosaic data enhancement, Mixup data enhancement, adaptive anchor frame calculation, adaptive image scaling, and other techniques. The backbone network module has a total of 50 layers. First, there are 4 convolutional layers or 4 CBS layers. Then, the data are processed through an ELAN module. ELAN consists of multiple CBS layers; the scale of input and output features remains the same, the number of channels differs in the first two CBS, the next few input channels are consistent with the output channels, and the last CBS output is the desired channel. Next are three MP-1 + ELANs. The MP-1 block is predominantly partitioned into Maxpool and CBS, and its output is one channel. Finally, three feature layers are formed through the output of the three MP-1 + ELAN modules. The depth characteristics obtained through the backbone network are input into the neck module. The entire neck module contains the SPPCPC layer, two MPConv layers, two UPSample layers, four ELAN-1 layers, four CBS layers, four CatConv layers, and three REPConv layers. The characteristics acquired by the backbone network can be obtained through a series of calculations of the neck module to obtain the ultimate result.

In the input module, Mosaic data enhancement merges four images by arbitrarily scaling, cropping, and arranging them to enrich the background of the detection object and improve the performance of small things. Mixup data enhancement combines two random samples proportionally, and the results of the classification are proportionally distributed. It can enhance the generalization ability of neural network architecture and reduce the memory of false labels. Adaptive anchor frame calculations enable the adaptive computation of the optimal anchor frame values in various training sets. Adaptive image scaling reduces the black bars to be filled after the image is scaled, and the quantity of computation is significantly reduced during inference so that the object detection performance is enhanced.

**Figure 3:**
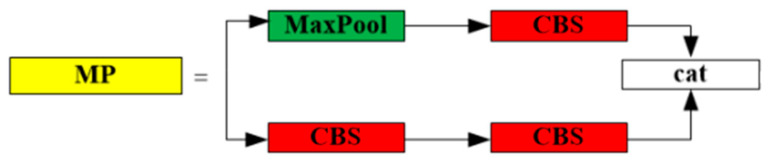
The MP Structure.

**Figure 4:**
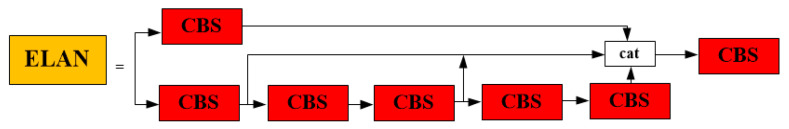
The ELAN structure.

In the backbone module, an MP structure is formed by maxpooling and cnov to improve the ability of the backbone module to extract features. As the convolutional neural network deepens, the information disappears or expands through many layers, and the shorter connection of the convolutional neural network in the layer close to the input or near the output significantly increases the depth of the model and at the same time greatly improves the efficiency of the training. Based on this idea, the ELAN structure was designed, enabling the backbone network to achieve an efficient aggregation network. The MP structure shows in Figure 3. The ELAN structure shows in Figure 4.

**Figure 5:**
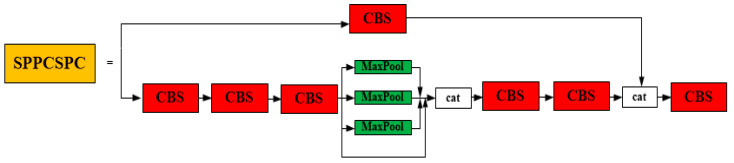
The SPPCSPC structure.

**Figure 6:**
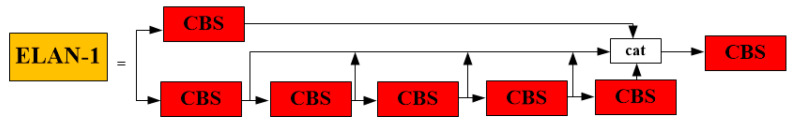
The ELAN-1 structure.

**Figure 7:**
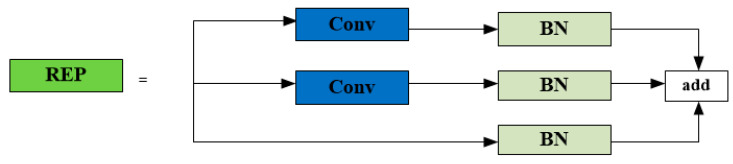
The REP structure.

In the neck module, the SPPCPC structure is the first space pyramid pooling structure proposed in YOLOv7. SPPCPC has a significant improvement compared with the previous SPP structure, but the amount of parameters and calculations is also much greater. After the ELAN structure was changed for the neck as ELAN-1, it was also applied in the neck module. The REP structure is a structural reparameterization. It is also an important improvement in the neck module of YOLOv7. It makes the YOLOv7 run faster without sacrificing precision. The SPPCPC structure shows in Figure 5. The ELAN-1 structure shows in Figure 6. The REP structure shows in Figure 7.

#### 2.3.2. Attention Mechanism

The human visual system can naturally find important positions in image information, and when the information of important positions is obtained, the recognition and judgment are greatly accelerated. Introducing this idea into computer vision, where the adaptive attention of convolutional neural networks is important, can improve the precision and speed of the YOLOv7. So far, the attention mechanism has developed a number of categories, among which channel attention mechanism, spatial attention mechanism, and the combination of the two-channel and spatial attention mechanisms are the more mainstream attention mechanisms.

Channel attention mechanism: The CNN features of a two-dimensional image usually have three dimensions: length, width, and channel. To improve the ability to represent the feature, the channel attention mechanism weighs the channels of the convolutional feature. The SEnet attention mechanism [26] is the pioneering work of the channel attention mechanism. The SEnet is divided into a squeeze module and an excitation module, which can collect the global information, capture channel relationships, and improve the ability to extract features. However, SEnet is unable to directly model the correspondence between the weight vector and the input, which reduces the quality of the results. The ECAnet attention mechanism [27] uses one-dimensional convolution to determine interactions between channels, rather than dimensionality reduction, to provide a fast, efficient module that can be easily inserted into various convolutional neural networks. Additionally, channel attention mechanisms are also included: GSoP [28], SRM [29], GCT [30], Fca, etc. [31].

Spatial attention mechanism: The STN attention mechanism [32] transforms the spatial information in the original image into another space through a spatial transformation and preserves the key information. Meanwhile, the pooling layer in the convolutional neural network uses maximum pooling or average pooling methods to compress the image information, reducing the amount of operation and improving the precision. The GEnet attention mechanism [33] can take advantage of the ability to recalibrate within the spatial domain to capture contextual information in remote space. The Vit attention mechanism [34] was first used for image processing as a pure converter architecture. It obtained results comparable to modern convolutional neural networks. In addition to the above, spatial attention mechanisms include DCN [35], PSAnet [36], SASA [37], and so on.

Channel and spatial attention mechanism: The channel and spatial attention mechanism combines the channel attention mechanism and the spatial attention mechanism. It includes the advantages of both. The CBAM attention mechanism [38] consists of two sub-modules, CAM and SAM, which perform channel and spatial attention, respectively. It not only saves the number of parameters and amount of computing power but also makes it easy to insert it into existing network architectures. In addition, channel space attention mechanisms also include CA [39], SCNet [40], SCA-CNN [41], scSE [42], Triplet Attention, etc. [43].

The SimAM attention mechanism [44] is a parameterized simple attention module based on neuroscience theory. When designing this attention mechanism, Lingxiao Yang’s team at Sun Yat-sen University considered both the channel attention mechanism and spatial attention mechanism and designed a parameterized 3D attention mechanism based on neuroscience theory. When attention mechanisms are at work, neurons are assigned weights based on their importance. Neurons have the property of spatial inhibition [45], i.e., more active neurons inhibit the activity of peripheral neurons. Moreover, the neurons that contain the most information in the optic nerve differ from the firing patterns of ordinary neurons. Based on the above two characteristics, the higher the inhibitory effect, the greater the importance of neurons. The linear separability of the target neuron from other neurons can be used to measure the energy of the target neuron, whereby the following energy functions can be defined:

(1) $$e_{t}(w_{t},b_{t},y,x_{i})=(y_{t}-\overset{\wedge }{t}{)}^{2}+\frac{1}{M-1}\sum_{i=1}^{M-1}{\left(y_{0}-{\overset{\wedge }{x}}_{i}\right)}^{2}$$

where $\hat{t}=w_{t}t+b_{t}$ and ${\hat{x}}_{i}=w_{t}x_{i}+b_{t}$ are linear transformations of t and $x_{i}$. t and $x_{i}$ represent the target neurons and other neurons of the input characteristics, respectively, and i is the index of the spatial dimension. M is the number of neurons in that channel. $w_{t}$ and $b_{t}$ represent the weights and biases of a neuron as it transforms, respectively. By minimizing this equation, we can obtain the linear severability of the target neuron and other neurons using binary labels instead of $y_{0}$ and $y_{t}$, adding regularization to the equation, and finally obtaining the energy equation:

(2) $$e_{t}(w_{t},b_{t},y,x_{i})=\frac{1}{M-1}\sum_{i=1}^{M-1}{\left(-1-(w_{t}x_{i}+b_{t})\right)}^{2}+{\left(1-(w_{t}t+b_{t})\right)}^{2}+\lambda w_{t}^{2}$$

Theoretically, each channel has M energy functions. The solution to Equation (2) is as follows:

(3) $$w_{t}=-\frac{2(t-\mu_{t})}{{\left(t-\mu_{t}\right)}^{2}+2\sigma_{t}^{2}+2\lambda }$$

(4) $$b_{t}=\frac{1}{2}(t+\mu_{t})w_{t}$$

In the formula, $\mu_{t}=\frac{1}{M-1}\sum_{i=1}^{M-1}x_{i}$ and $\sigma_{t}^{2}=\frac{1}{M-1}{\sum_{i}^{M-1}\left(x_{i}-\mu_{t}\right)}^{2}$ represent the standard deviation and variance of all other neurons in this channel except the target neuron *t*, respectively. Substituting $w_{t}$ and $b_{t}$ into Equation (2), respectively, yields the minimum energy.

(5) $$e_{t}^{*}=\frac{4({\hat{\sigma }}^{2}+\lambda )}{{\left(t-\hat{\mu }\right)}^{2}+2{\hat{\sigma }}^{2}+2\lambda }$$

In the formula, for $\mu =\frac{1}{M}\sum_{i=1}^{M-1}x_{i}$ and $\sigma^{2}=\frac{1}{M}{\sum_{i}^{M-1}\left(x_{i}-\mu_{t}\right)}^{2}$, $\frac{1}{e_{t}^{*}}$ can be used to represent the importance of neurons.

The regulation of attention mechanisms in the mammalian brain manifests itself as scaling to neurons, so we used scaling operators for feature optimization.

Based on this, this paper introduces the attention mechanism into the YOLOv7 object detection model to obtain a faster and more precise object detection model. In this paper, we selected five attention mechanisms to introduce into YOLOv7, trained them separately on the cracks dataset we collected, obtained their respective object detection models, and then screened the performance of the models for best results.

#### 2.3.3. Design Method

The design idea of the backbone network part of YOLOv7 is already excellent. Inserting attention mechanisms into backbones can disrupt the integrity of backbones, resulting in training that is less effective than before. Between the backbone structure and the neck structure of YOLOv7, there are three feature layers of connection. We insert attention mechanisms here.

The network architecture process of the model: The image is input to the backbone network. According to the output of the three layers in the backbone network, the features are refined by the attention mechanism, the feature map with different sizes is continued to be output in the head layer through the backbone network in three layers, and the final result is output after the RepVGG block and conv for image prediction.

In this study, YOLOv7 was used as a baseline to insert different attention mechanisms to improve and construct rock crack recognition technology based on deep learning. To increase the credibility of the model experiments, this study used YOLOv5 as a benchmark for comparison.

### 2.4. Performance Metrics

There are four things that can happen to the results in object detection: *TP* indicates that the prediction is positive and the label is positive; *FP* indicates that the prediction is positive and the label is negative; *FN* indicates that the prediction is negative and the label is positive; and *TN* indicates that the prediction is negative and the label is negative.

Criteria for object detection models include *p*recision, re*call*, average precision, etc.

**Precision** indicates that the label is positive and accounts for the proportion of all results predicted as positive.

(6) $$P\mathrm{recision}=\frac{TP}{TP+FP}$$

**Recall** indicates that the result predicted is positive and accounts for the proportion of all results labeled as positive.

(7) Recall=TPTP+FN

**Average Precision**: *P*recision and re*call* are contradictory in some extreme cases, so it is necessary to draw a PR curve to judge. Simply put, it is the mean of the precision value on the PR curve. For PR curves, the integral can be used for calculations, and the resulting value is the mean precision (AP, average precision).

**Time100** indicates the time required to process 100 images.

## 3. Results and Analysis

### 3.1. Parameters and Calculation

Param shows the value of the model parameters, and FLOPs shows the amount of computations for the model. The amount of model parameters and calculations determine the efficiency of the model training. The amount of model parameters and calculations is introduced in Table 1.

It can be seen that the SimAM attention mechanism does not increase the number of parameters after the insertion of YOLOv7, while other attention mechanisms increase the model parameters to varying degrees. The increase in the number of parameters reduces the efficiency of the model to some extent when training. The attention mechanism increases the number of parameters of the model while inserting the model, which inevitably reduces the computational efficiency of the model. As a parameterless attention mechanism, SimAM avoids this problem.

### 3.2. Training Loss

During model training, the loss of the model decreases with training. The loss reduction in each attention mechanism combined with the YOLOv7 model is shown in Figure 1. Table 2 and Table 3 introduce the total loss and val loss, respectively.

**Figure 8:**
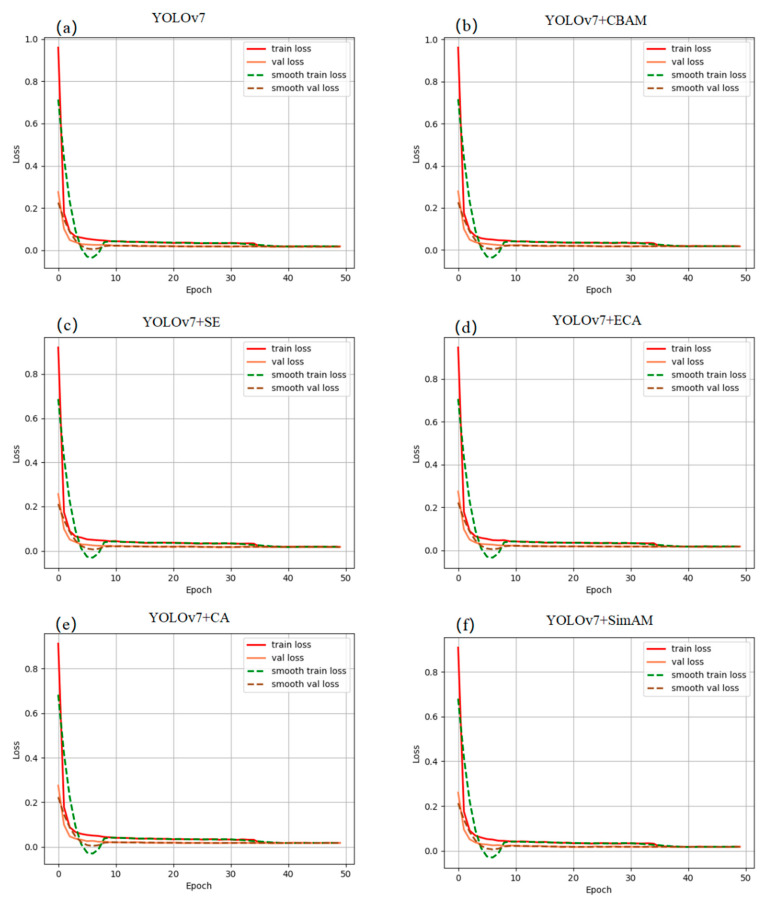
Loss of different models. (**a**) YOLOv7, (**b**) YOLOv7 + CBAM, (**c**) YOLOv7 + SE, (**d**) YOLOv7 + ECA, (**e**) YOLOv7 + CA, (**f**) YOLOv7 + SimAM.

Figure 8 and Table 2 and Table 3 show the loss drop during the training of each model, including total loss and val loss. With the help of various attention mechanisms, the rate of loss decline in other models improved to varying degrees. According to Table 2, the SimAM attention mechanism has a rapid rate of loss decline in the early stage of training, and the loss curve quickly converges to reach a stable state. According to Table 3, the SimAM attention mechanism decreases faster than other attention mechanisms at the beginning of training and achieves the best loss rate at the end.

### 3.3. Performance

**Figure 9:**
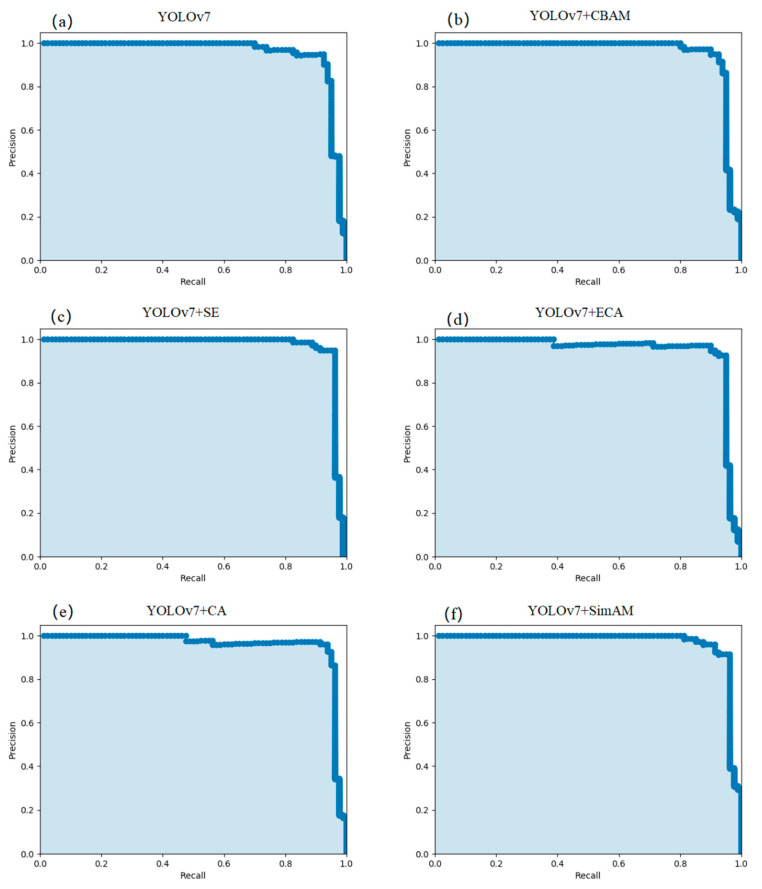
PR Curve of different models. (**a**) YOLOv7,(**b**) t YOLOv7 + CBAM, (**c**) YOLOv7 + SE, (**d**) YOLOv7 + ECA, (**e**) YOLOv7 + CA, (**f**) YOLOv7 + SimAM.

After training, the training parameters of each model can be obtained. The parameters can be used for the object detection test model. Performance metrics for object detection include precision, recall, average precision, and Time100. The PR curve of each model is shown in Figure 9, and the detection performance of each model is shown in Table 4.

The performance of each model is shown in Figure 9 and Table 4. The baseline model, YOLOv7, reached 98.33% in terms of precision, and the models that incorporate the CBAM attention mechanism, the SE attention mechanism, and the SimAM attention mechanism increased the precision of the model to 100%. ECA and CA combined with YOLOv7 have a certain degree of reduction in precision, attaining only 96.83% and 96.61%, respectively.

The recall rate of the baseline model, YOLOv7, was 73.75%. The improvement in the ECA attention mechanism after insertion into the original model reached 76.25%, followed by the SimAM attention mechanism, which achieved 75% after insertion of the recall rate, and the CBAM attention mechanism and the original model, YOLOv7, were the same. However, the CA attention mechanism and the SE attention mechanism led to a decrease in the recall rate of the model, of 71.25% and 68.75%, respectively.

The average precision combined precision and recall rate can better demonstrate the performance of the model, for which the SimAM attention mechanism after insertion into YOLOv7 improved the degree of the maximum degree of the model’s AP from 95.44% to 96.89%, an increase of 1.45%. Except for the ECA attention mechanism, which lowered the AP of the model, the other attention mechanisms improved it to varying degrees.

## 4. Discussion

Comparing the six models in this study, a comprehensive view shows that YOLOv5 has the worst performance compared to the other models. This study inserted five different attention mechanisms between the backbone module and the neck module of the YOLOv7 object detection model, which affected the efficiency of the object detection model to varying degrees. The results show that the biggest performance improvement in the model was the SimAM attention mechanism. It did not increase the number of parameters of the model when inserting the model, and at the same time, it improved the performance of the model to the greatest extent compared with the other four attention mechanisms overall. Arunabha et al. proposed DenseSPH-YOLOv5 based on YOLOv5 for improvement by CBAM, additional feature fusion layers, and a Swin-Transformer Prediction Head (SPH) [46]. Ziang Cao et al. combined the CBAM attention mechanism with YOLOv5 for persimmon detection, with a precision of 92.69% and an AP of 95.53%, which was 1.51% and 0.63% higher than the original model [47], and the increase was more consistent with the results of this study. The spatial attention map of the CBAM attention mechanism was generated using convolution, so the attention mechanism part is affected by the receiving field to a certain extent, and its insertion increases the most parameters. In this study, the combination of SEnet and YOLOv7 improved the precision by 1.67% and the AP by 0.77%, compared to the original model’s precision. Zhenrong Deng et al. combined the YOLOv3 model with improved Anchor Box Algorithms and SENet attention mechanisms for small-scale face detectors for outdoor security [48]. The new model is 2.2% better in precision and 1.4% better than the original model. The SE attention mechanism cannot directly model the correspondence between the weight vector and the input, which reduces the quality of the results to a certain extent. The ECA attention mechanism module uses a 1 × 1 convolutional layer directly after the global average pooling layer, removing the fully connected layer. In this paper, it increased the speed of the object detection model to some extent, but the precision did decrease to a certain extent. Yuanyang Cao et al. combined the ECA attention mechanism with YOLOv5x to apply dynamic sheep counting [49]. Compared with the original model, the precision of the model was improved by 0.76%, which is similar to the results of this study. On the one hand, the datasets are different, and on the other hand, the different locations introduced by the attention mechanism cause the difference in results. The CA attention mechanism decreased rapidly during training, but the precision effect was not good when using the trained parameters for object detection.

This study merely used a model derived by inserting five attention mechanisms into the same position of the original model. Because the design concepts, mechanisms, and principles of various attention mechanisms are different, their characteristics are also different. Therefore, the insertion location and the trained dataset also had a significant impact on the efficacy of the model. Based on the above, the next study will evaluate the position of the attention mechanism insertion, the combination of attention mechanisms, and the impact of various datasets on the performance of the object detection model to obtain a superior rock mass crack object detector.

## 5. Conclusions

The YOLOv7 object detection model surpasses the more widely used YOLOv5 object detection models in both precision and speed. Attention mechanisms make convolutional neural networks focus on areas that need attention, improve the performance of trained models, and increase the speed and precision of detecting models. Based on this, we inserted five attention mechanisms, CBAM, SE, ECA, CA, and SimAM, into YOLOv7. We compared the performance of these models to select the best attention mechanism of these five. In order to obtain the best rock crack object detection model, we combined the best attention mechanism with YOLOv7.

After comparison, it is evident that SimAM is the finest attention mechanism. First of all, the quantity of parameters and calculations did not increase. This means that the burden of training and detection did not increase. Secondly, the loss of the training model accelerated, and the loss curve rapidly converged. This implies that the pace of the training of the model was more rapid than before. Then, the precision reached up to 100%, the recall was second only to ECA, and the average precision reached a maximum of 96.89% in several attention mechanisms. Taken together, SimAM has a tremendous advantage over the other attention mechanisms. In summary, this study integrated the SimAM attention mechanism with the YOLOv7 object detection model to form an enhanced object detection model. In this study, the enhanced YOLOv7 object detection model was applied for the first time in the identification of rock mass crocks, an early symptom of geological catastrophes. The model attained excellent performance, and the object detection method for cracks in rock mass, an early indicator of geological disasters, can be obtained.

At present, the object detection method is relatively perfect. Few studies have used deep learning to identify early signs of disasters. In this study, an improved YOLOv7 object detection method based on the attention mechanism was applied for the first time to the identification of rock cracks in geohazards. Rock crack recognition technology based on deep learning is faster and more precise in discerning the presence and location of fractures in images. In the current study, there is still more research space for more refined and quantified measurements of early signs of geological disasters. If the development pattern of fissures needs to be studied more deeply, the calculation of parameters such as fissure width, length, and area can be realized in combination with the function of image segmentation.

## Figures and Tables

**Table 1:** Parameters and calculation of different models.

Model	Size	#Param.	FLOPs
Benchmark	640	47.054 M	115.648 G
Baseline	640	37.620 M	106.472 G
Baseline + CBAM	640	38.210 M	106.487 G
Baseline + SE	640	37.915 M	106.483 G
Baseline + ECA	640	37.620 M	106.483 G
Baseline + CA	640	38.063 M	106.514 G
Baseline + SimAM	640	37.620 M	106.472 G

**Table 2:** Total loss of different models.

Model	Epoch1	Epoch10	Epoch20	Epoch30	Epoch40	Epoch50
Benchmark	1.9423	0.0837	0.0693	0.0572	0.0371	0.0382
Baseline	0.9596	0.0427	0.0354	0.0320	0.0176	0.0184
Baseline + CBAM	0.9611	0.0433	0.0343	0.0342	0.0191	0.0175
Baseline + SE	0.9203	0.0434	0.0369	0.0335	0.0180	0.0182
Baseline + ECA	0.9469	0.0422	0.0357	0.0342	0.0185	0.0178
Baseline + CA	0.9119	0.0427	0.0362	0.0333	0.0178	0.0182
Baseline + SimAM	0.9040	0.0423	0.0339	0.0336	0.0191	0.0184

**Table 3:** Val loss of different models.

Model	Epoch1	Epoch10	Epoch20	Epoch30	Epoch40	Epoch50
Benchmark	0.8399	0.0401	0.0311	0.0284	0.0290	0.0294
Baseline	0.2758	0.0237	0.0196	0.0173	0.0174	0.0172
Baseline + CBAM	0.2782	0.0220	0.0190	0.0186	0.0177	0.0176
Baseline + SE	0.2562	0.0227	0.0191	0.0174	0.0172	0.0167
Baseline + ECA	0.2740	0.0220	0.0179	0.0179	0.0174	0.0173
Baseline + CA	0.2753	0.0208	0.0187	0.0177	0.0168	0.0176
Baseline + SimAM	0.2619	0.0232	0.0188	0.0181	0.0173	0.0163

**Table 4:** Performance of different models.

Model	Precision	Recall	AP	F1	Time100 (s)
Benchmark	95.7%	55.16%	88%	0.81	12
Baseline	98.33%	73.75%	95.44%	0.84	10
Baseline + CBAM	100%	73.75%	95.68%	0.85	34
Baseline + SE	100%	68.75%	96.21%	0.81	31
Baseline + ECA	96.83%	76.25%	94.60%	0.85	16
Baseline + CA	96.61%	71.25%	95.68%	0.82	11
Baseline + SimAM	100%	75%	96.89%	0.86	10

## Data Availability

Not applicable.
